# First detection of N1575Y mutation in pyrethroid resistant *Anopheles gambiae* in Southern Côte d’Ivoire

**DOI:** 10.12688/wellcomeopenres.12246.1

**Published:** 2017-08-24

**Authors:** Ako Victorien Constant Edi, Bedjou Prisca N'Dri, Mouhamadou Chouaibou, Fondjo Behi Kouadio, Patricia Pignatelli, Giovanna Raso, David Weetman, Bassirou Bonfoh

**Affiliations:** 1 Research and Development Department, Centre Suisse de Recherches Scientifiques en Côte d’Ivoire, Abidjan, 01 BP 1303, Cote d'Ivoire; 2 Swiss Tropical and Public Health Institute, Basel, CH-4051 , Switzerland; 3 University of Basel, Basel, CH-4002 , Switzerland; 4 Vector Biology Department, Liverpool School of Tropical Medicine, Liverpool, , L3 5QA, UK

**Keywords:** Insecticide resistance, deltamethrin, N1575 Y mutation, Anopheles coluzzii, Anopheles gambiae, Côte d’Ivoire, Tiassalé, Elibou

## Abstract

**Background.** The intensification of insecticide use for both public health and agriculture in Africa has contributed to growing insecticide resistance. Today, resistance to World Health Organization (WHO)-approved insecticide classes is widespread. In an agricultural area of Southern Côte d’Ivoire, the main malaria vector *Anopheles coluzzii* shows multiple resistance across insecticides mediated by both target site mutation and metabolic mechanisms. To plan new vector control strategies and avert future resistance liabilities caused by cross-resistance mechanisms extant within populations, it is crucial to monitor the development and spread of both resistance and mechanisms.

**Methods.**  Larvae of *Anopheles gambiae* were collected from natural breeding sites in Tiassalé and Elibou, between April and November 2016 and raised to adults **.** Adult female non-blood fed mosquitoes, three to five days old, were exposed to deltamethrin in WHO bioassays. Extracted DNA samples from exposed mosquitoes were used for species characterisation and genotyping.

**Results.** Most adult *An. gambiae* tested were resistant to deltamethrin, with mortality rates of only 25% in Tiassalé and 4.4% in Elibou. Molecular analysis of DNA from samples tested showed the presence of both *An. coluzzii* and *An. gambiae s.s* in Elibou and only *An. coluzzii* for Tiassalé. As previously, the L1014F *kdr *mutation was present at high frequency (79%) in Tiassalé and the L1014S mutation was absent. The N1575Y mutation, which amplifies resistance conferred by L1014F was detected in a single unique individual from a Tiassalé *An. coluzzii* female whereas in Elibou 1575Y was present in 10 *An. gambiae* s.s, but not in *An. coluzzii*.

**Conclusion.** This is the first report of the N1575Y mutation in Côte d’Ivoire, and as in other populations, it is found in both dominant West African malaria vector species. Continued monitoring of N1575Y is underway, as are studies to elucidate its contribution to the resistance of local vector populations.

## Introduction

Malaria remains an important tropical disease that requires a global effort for eradication ( WHO malaria report, 2016). Scale-up of existing control measures and development of new tools for the markets (2016–2030) are key components of the global control and elimination strategies ( WHO Global Malaria programme, 2016). The development of new tools is of utmost importance, since resistance in the major *Anopheles* malaria vectors of sub-Saharan Africa to WHO-approved insecticides is now widespread and increasing ( Ranson & Lissenden, 2016). Increasingly, populations of mosquitoes that are resistant to more than one class of insecticides are being reported ( Djouaka *et al.*, 2016; Edi *et al.*, 2014a; Nwane *et al.*, 2013).

Among insecticides, pyrethroids remain the only class approved for long-lasting insecticide treated nets, and major resistance mechanisms are metabolic detoxification, especially by P450 enzymes ( David *et al.*, 2013), and mutations in the *para* voltage gated sodium channel (VGSC), the target site of pyrethroids and DDT. VGSCs are transmembrane proteins that transfer sodium ions inside the cell in order to achieve the depolarizing phase of action potentials, an essential phase of nervous impulses ( Catterall *et al.*, 2005). Mutations in the VGSC cause a phenotype known as knock down resistance ( *kdr*). The most common *kdr* mutations in *Anopheles* are substitutions at the 1014 leucine codon to either phenylalanine ( Martinez Torres *et al.*, 1998) or serine ( Ranson *et al.*, 2000). Both are now widely distributed across Africa, and sometimes co-occur ( Fryxell *et al.*, 2012; Nwane *et al.*, 2011; Pinto *et al.*, 2006; Reimer *et al.*, 2008; Tripet *et al.*, 2007). Moreover, their frequency could differ from a mosquito population tested with insecticide to another ( Antonio-Nkondjio *et al.*, 2015). An additional asparagine-to-tyrosine mutation at codon 1575 within the linker between domains III-IV of the VGSC has also been documented in *An. gambiae* and/or *An. coluzzii* from Burkina Faso, Ghana, Benin and Cameroon ( Jones *et al.*, 2012; Fossog Tene *et al.*, 2013). The N1575Y mutation provides a synergistic effect on pyrethroid and DDT resistance by elevating the insensitivity of the sodium channel gates produced by the 1014F and 1014S mutations ( Wang, 2013), although to date it has only been found on the 1014F haplotype ( Jones *et al.*, 2012).

Vector control requires improved management of insecticides and identification of mechanisms of resistance, which can be readily screened to forewarn increases in resistance or represent possible cross-resistance liabilities to new insecticides when they become available. Previous studies in Southern Cote d’Ivoire have documented resistance to multiple insecticides, mediated by multiple mechanisms ( Edi *et al.*, 2012; Edi *et al.*, 2014a); however, all samples screened were wild type at the 1575 codon ( Edi *et al.*, 2012). Here we report the first detection of N1575Y mutation in deltamethrin resistant populations of *An. gambiae* and *An. coluzzii* from Southern Côte d’Ivoire. 

## Methods

### Study site

**Figure 1.**
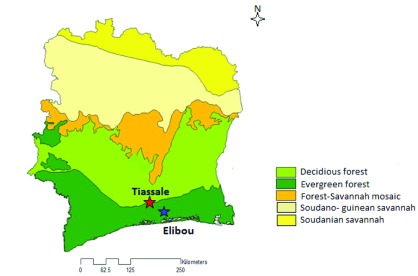
Vegetation map of Côte d’Ivoire, showing the study areas Tiassalé and Elibou.

All collections were carried out in Tiassalé (latitude 5.89839, longitude -4.82293) and Elibou (latitude 5.69000, longitude - 4.50000), Southern Côte d’Ivoire ( Figure 1). The study sites are located in the evergreen forest zone. The primary agricultural activity is cash crop farming of vegetables in Elibou and irrigated rice fields in Tiassalé. High malaria transmission occurs during the rainy seasons, between May and November.

### Larval collections

Larval collections of *An. gambiae s.l.* were obtained by dipping from breeding sites in each study location during the peak malaria transmission periods between April and November 2016 **.** All larvae were provided a diet of Tetra Mikromin fish food (Tetra; Melle, Germany) until adult emergence. All mosquito rearing was performed under controlled ambient environmental conditions (27°C±2°C, 80% ±4% relative humidity). Adult mosquitoes were given access to 10% sucrose solution.

### WHO diagnostic bioassays

Adult female non-blood fed mosquitoes, two to five days old, were exposed to 0.05% deltamethrin for one hour, using WHO tubes and criteria ( WHO, 2013). Mosquitoes surviving insecticide exposure were given access to 10% sucrose solution in WHO holding tubes. Mortality was assessed after 24 hours. For each replicate, fifty mosquitoes were exposed to non-treated filter papers as a control in two separate tubes. Exposed mosquitoes were then stored with silica gel until DNA extraction. Levels of mortality between study locations were compared using Chi-square test run on the open source software package R, version 3.4.0 ( R Core Team, 2008). For statistical testing, the level of significance was set at α = 0.05.

### DNA extraction

Genomic DNA was extracted according to the LIVAK method ( Livak, 1984) from individual *An. gambiae s.l.* mosquitoes from Tiassalé (n=92) and Elibou. In Tiassalé, extraction was made on individuals surviving exposure to deltamethrin. In Elibou, additional 110 untested females (control samples) obtained from larval collection were considered. Mosquitoes were individually ground in 100µl of preheated grind buffer made with 1.6 ml 5M Nacl, 5.48 g sucrose, 1.57g Tris, 10.16 ml 0.5M EDTA and 2.5 ml 20% SDS (Thermo Fisher Scientific). Mixed buffer-mosquito solution was incubated at 65°C for 30 minutes and then 14 µl 8M K-acetate was incorporated and gently mixed. The resulting thicker mixture was incubated for 30 min on ice and then centrifuged at 13,000 rpm for 20 min (4°C). 200 µl 100% EtOH was then added to the supernatant (later transferred in new Eppendorf tube 1.5 ml) and the mixture was centrifuge again at 13,000 rpm for 15 min (4°C). The supernatant was discarded and the pellet was rinsed with 100 µl ice cold 70% EtOH. Dried pellets were re-suspended in 100 µl TAE buffer. The DNA was used for species ID and target site genotyping.

### Species and molecular form identification

The SINE-PCR method was used to identify *An. gambiae s.l.* to species ( Santolamazza *et al.*, 2008). A volume of 24.75 µl of master mix was considered per reaction. Overall the master mix (Applied Biosystems) contained 18.83 µl DNase free water, 2.5 µl buffer 10X, 0.75 µl MgCl2 (25mM), 1 µl dNTP (10mM) and respective 1 µl of Sine 6.1a (10 µM) 5’-CGCTTCAAGAATTCGAG ATAC-3’ and Sine 6.1b (10 µM) 5’-TCGCCTTAGA CCTTGCGTTA-3’and 0.17 µl Kappa Taq. Each PCR product containing 23 µl of mix and 2 µl of genomic DNA was amplified for 3 min at 94°C, followed by 35 cycles of 94°C, 62°C, and 72°C for 30 s respectively. The last cycle of was 5 min at 72°C. Products were run on 1.5% agarose gels. In Elibou, untested female samples were used for species and molecular form identification. In Tiassalé, only individual surviving exposure to deltamethrin were considered.

### Genotyping assays

TaqMan assays with two labelled Fluorochromes probes FAM and HEX were used to screen for the L1014F and L1014S *kdr* mutations ( Bass *et al.*, 2007) and the N1575Y mutation ( Jones *et al.*, 2012). A total volume of 9 µl per reaction was used for the mix, containing DNase free water (3.875 µl), Bioline sensimix (5 µl) and specific primer/probe (0.125 µl) ( *kdr*-Forward 5'-CATTTTTCTTGGCCACTGTAGTGAT-3', *kdr*-Reverse 5'-CGATCTTGGTCCATG TTAATTTGCA-3') for *kdr* 1014 and (3′NFQ-ATTTTTTTCATTGCATTATAGTAC-5’ for N1575 and 3′NFQ-TTTTTCATTGCATAATAGTAC-5’for 1575Y, respectively. The mix was centrifuged at 2000 rpm for approximately 10 seconds. 9 µl of the mix with 1 µl of each gDNA were added to each TaqMan PCR (Applied Biosystems), and centrifuged at 2000 rpm for 15 seconds. Reactions were performed on the Agilent MX3005P qPCR system (Agilent Technologies). The genotype was determined from the fluorescence profiles and bi-directional scatter plots generated in the MX3005P software. The PCR condition was 95°C for 10 minutes (1 cycle) following by 40 cycles of 95°C at 10 seconds and 60°C at 45 seconds, respectively for Kdr genotyping. For N1575 Y mutation, PCR conditions of 10 min at 95°C followed by 40 cycles of 15 s at 92°C and 1 min at 60°C were considered ( Jones *et al.*, 2012).

## Results

A total of 291 mosquitoes were tested with deltamethrin in Tiassalé (n=200) and Elibou (n=91) during the rainy season, using standard WHO susceptibility assays. Mortality rates were 25% in Tiassalé and 4.4% in Elibou. The prevalence of *An. gambiae* resistance was different between study locations (Chisq=13, p=0.0003).

All mosquitoes were collected at larval stage. In Elibou, the first emerged (n= 91) were used for bioassay, as previously described, and a sample of 110 from the remaining untested ones were used for molecular form and species identification. All these mosquitoes were from the same batch. Thus, from a subset of DNA samples analysed in Elibou (n= 110) and Tiassalé (n=92), no *An. arabiensis* were detected. All individuals were found to be *An. coluzzii* (100%) in Tiassalé. In Elibou, both *An. coluzzii* (53.6%, n= 59) and *An. gambiae* ss (35.5%, n= 39) and additional hybrid individuals (10.9%, n= 12) were present.

**Figure 2.**
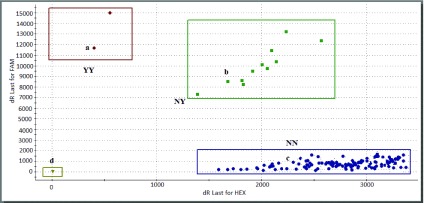
Distribution of N1575Y alleles in Southern Côte d’Ivoire. The letters a, b, c and d represent the positive controls for the homozygous mutant allele (Y), heterozygous mutant (NY) and the homozygous susceptible allele (N) and blank, respectively. Nine individual mosquitoes displayed the heterozygous mutant/susceptible genotype (NY), only one individual carried the homozygous resistant genotype (YY). All the other samples carried the susceptible genotype (NN).

Of the two potential substitutions screened at codon 1014, 1014S was absent, whereas the 1014F *kdr* allele was observed in Tiassalé at high frequency (79.3%). In Elibou, the frequencies of L1014 mutation were not investigated. From the 92 surviving *An. coluzzii* mosquitoes exposed to deltamethrin, a single individual was found to carry the N1575Y mutation (in heterozygous form) in Tiassalé ( Table 1). In Elibou, both heterozygous (20.4%) and a single homozygous resistant individual (2.2%) were detected in *An. gambiae* s.s ( Figure 2). The overall 1575Y allele frequency in Elibou was 25% ( Table 1).

**Table 1.** Prevalence of N1575Y allele in Tiassalé and Elibou, Côte d’Ivoire, 2016.

Strains	Species	Phenotype	No. tested	No. per genotype			Frequency (%)
				NN	NY	YY	
Tiassalé	*An. coluzzii*	Alive	92	91	1	0	0.5
Elibou	*An. coluzzii*	Alive	22	22	0	0	0
	*An. gambiae s.s*	Alive	22	12	9	1	25

Y and N represent mutant resistant (tyrosine) and wild types alleles (asparagine), respectively.

First detection of N1575Y mutation in pyrethroid resistant Anopheles gambiae in Southern Côte d’Ivoire **Dataset 1: **Raw data for mortality rates from WHO bioassays in Tiassalé.
**Dataset 2: **Raw data for mortality rates from WHO bioassays in Elibou.
**Dataset 3: **Raw data for N1575Y genotypes. Click here for additional data file. Copyright: © 2017 Edi AVC et al. 2017

## Discussion

The aim of this study was to investigate the level of resistance to the pyrethroid deltamethrin in Southern Cote d’Ivoire. It was found that the prevalence of *An. gambiae* resistance was different between study locations. In Tiassalé, the mortality rate to deltamethrin was 25%, while this was six-folds lower in Elibou. Resistance to deltamethrin in the Southern region could be explained by the insecticide pressure due to development of agriculture and use of insecticide in the region Reid & McKenzie, 2016. Indeed, deltamethrin insecticides are largely used by farmers for both pest control and increase of yield. A recent finding also showed that among the main pyrethroid insecticides used in Tiassalé, deltamethrin was the most common (46.9%) followed by lambdacyhalothrin (35.2%) and cypermethrin (10.5%) ( Chouaïbou *et al.*, 2016). To our knowledge, there is no cultivation in the Elibou village, but in the surroundings, there is intense rubber (hevea) farming, where insecticides are also applied. Thus, potential contamination of existing breeding sites could occur given the ecology of the region (see description of study sites), and further investigations are required to better understand the emergence of resistance to deltamethrin in this area.

In Tiassalé, the presence of the 1575Y resistance allele was detected for the first time. This is surprising because previous consecutive data from 2011 (0%, n= 184) and 2012 (0%, n=92) did not report the N1575Y mutation ( Edi *et al.*, 2012; Edi *et al.*, 2014b). In Elibou, our finding revealed the presence of both heterozygous and homozygous resistant tyrosine alleles in *An. gambiae s.s*. Elibou is located within a 100 km radius from Tiassalé and characterized by the presence of both *An. coluzzii* and *An. gambiae s.s*. The detection of N1575Y mutation in *An*. *coluzzii* in Tiassalé and *An. gambiae s.s* in Elibou revealed the potential for both species to carry the asparagine-to-tyrosine resistance mechanisms, as previously documented in Burkina Faso ( Jones *et al.*, 2012).

The detection of N1575Y mutation now in southern Côte d’Ivoire requires more investigation to better characterize its expected synergistic relationship with 1014F *kdr*. This resistance mechanism could spread very rapidly, and threaten the fragile gains that have been made in reducing the malaria burden in this region through vector control interventions. 

## Conclusion

The present study showed the presence of the N1575Y mutation in Côte d’Ivoire. The discovery of an additional mechanism that could further reduce insecticide efficacy in the already pyrethroid resistant mosquitoes in this region is concerning. Continued monitoring of N1575Y is underway to elucidate its contribution to the resistance of local vector populations.

## Data availability

The data referenced by this article are under copyright with the following copyright statement: Copyright: © 2017 Edi AVC et al.

**First detection of N1575Y mutation in pyrethroid resistant *Anopheles gambiae* in Southern Côte d’Ivoire**

**Dataset 1:** Raw data for mortality rates from WHO bioassays in Tiassalé.

**Dataset 2:** Raw data for mortality rates from WHO bioassays in Elibou.

**Dataset 3:** Raw data for N1575Y genotypes.

https://doi.org/10.6084/m9.figshare.5319250.v1 ( Edi *et al.*, 2017)

## References

[ref-1] Antonio-Nkondjio C Tene Fossog B Kopya E: Rapid evolution of pyrethroid resistance prevalence in *Anopheles gambiae* populations from the cities of Douala and Yaoundé (Cameroon). *Malar J.* 2015;14:155. 10.1186/s12936-015-0675-6 25879950 PMC4403825

[ref-2] Bass C Nikou D Donnelly MJ: Detection of knockdown resistance ( *kdr*) mutations in *Anopheles gambiae*: a comparison of two new high-throughput assays with existing methods. *Malar J.* 2007;6:111. 10.1186/1475-2875-6-111 17697325 PMC1971715

[ref-3] Catterall WA Goldin AL Waxman SG: International Union of Pharmacology. XLVII. Nomenclature and structure-function relationships of voltage-gated sodium channels. *Pharmacol Rev.* 2005;57(4):397–409. 10.1124/pr.57.4.4 16382098

[ref-4] Chouaïbou MS Fodjo BK Fokou G: Influence of the agrochemicals used for rice and vegetable cultivation on insecticide resistance in malaria vectors in southern Côte d’Ivoire. *Malar J.* 2016;15(1):426. 10.1186/s12936-016-1481-5 27553959 PMC4995742

[ref-5] David JP Ismail HM Chandor-Proust A: Role of cytochrome P450s in insecticide resistance: impact on the control of mosquito-borne diseases and use of insecticides on Earth. *Philos Trans R Soc B Biol Sci.* 2013;368(1612): 20120429. 10.1098/rstb.2012.0429 23297352 PMC3538419

[ref-6] Djouaka RJ Atoyebi SM Tchigossou GM: Evidence of a multiple insecticide resistance in the malaria vector *Anopheles funestus* in South West Nigeria. *Malar J.* 2016;15(1):565. 10.1186/s12936-016-1615-9 27876039 PMC5120565

[ref-7] Edi CV Djogbénou L Jenkins AM: *CYP6 P450* enzymes and *ACE-1* duplication produce extreme and multiple insecticide resistance in the malaria mosquito *Anopheles gambiae*. *PLoS Genet.* 2014a;10(3):e1004236. 10.1371/journal.pgen.1004236 24651294 PMC3961184

[ref-10] Edi CA Koudou BG Bellai L: Long-term trends in *Anopheles gambiae* insecticide resistance in Côte d’Ivoire. *Parasit Vectors.* 2014b;7:500. 10.1186/s13071-014-0500-z 25429888 PMC4269959

[ref-8] Edi CV Koudou BG Jones CM: Multiple-insecticide resistance in *Anopheles gambiae* mosquitoes, Southern Côte d’Ivoire. *Emerg Infect Dis.* 2012;18(9):1508–1511. 10.3201/eid1809.120262 22932478 PMC3437712

[ref-9] Edi AV N'Dri BP Chouaibou M: First detection of N1575Y mutation in pyrethroid resistant *Anopheles gambiae* in Southern Côte d’Ivoire. *Figshare.* 2017 Data Source 10.12688/wellcomeopenres.12246.1 PMC5627500 29018842

[ref-11] Fossog Tene B Poupardin R Costantini C: Resistance to DDT in an urban setting: common mechanisms implicated in both M and S forms of *Anopheles gambiae* in the city of Yaoundé Cameroon. *PLoS One.* 2013;8(4):e61408. 10.1371/journal.pone.0061408 23626680 PMC3634070

[ref-12] Fryxell RT Seifert SN Lee Y: The knockdown resistance mutation and knockdown time in *Anopheles gambiae* collected from Mali evaluated through a bottle bioassay and a novel insecticide-treated net bioassay. *J Am Mosq Control Assoc.* 2012;28(2):119–22. 10.2987/11-6216R.1 22894124

[ref-13] Jones CM Liyanapathirana M Agossa FR: Footprints of positive selection associated with a mutation ( *N1575Y*) in the voltage-gated sodium channel of *Anopheles gambiae*. *Proc Natl Acad Sci U S A.* 2012;109(17):6614–9. 10.1073/pnas.1201475109 22493253 PMC3340067

[ref-14] Livak KJ: Organization and mapping of a sequence on the *Drosophila melanogaster* X and Y chromosomes that is transcribed during spermatogenesis. *Genetics.* 1984;107(4):611–34. 6430749 10.1093/genetics/107.4.611 PMC1202380

[ref-15] Martinez-Torres D Chandre F Williamson MS: Molecular characterization of pyrethroid knockdown resistance ( *kdr*) in the major malaria vector *Anopheles gambiae s.s*. *Insect Mol Biol.* 1998;7(2):179–184. 10.1046/j.1365-2583.1998.72062.x 9535162

[ref-16] Nwane P Etang J Chouaїbou M: Multiple insecticide resistance mechanisms in *Anopheles gambiae* s.l. populations from Cameroon, Central Africa. *Parasit Vectors.* 2013;6(1):41. 10.1186/1756-3305-6-41 23433176 PMC3583743

[ref-17] Nwane P Etang J Chouaїbou M: *Kdr*-based insecticide resistance in *Anopheles gambiae* s.s populations in Cameroon: spread of the L1014F and L1014S mutations. *BMC Res Notes.* 2011;4:463. 10.1186/1756-0500-4-463 22035176 PMC3221647

[ref-18] Pinto J Lynd A Elissa N: Co-occurrence of East and West African *kdr* mutations suggests high levels of resistance to pyrethroid insecticides in *Anopheles gambiae* from Libreville, Gabon. *Med Vet Entomol.* 2006;20(1):27–32. 10.1111/j.1365-2915.2006.00611.x 16608487

[ref-19] R Core Team: R: A language and environment for statistical computing. Vienna, Austria: R Foundaton for Statistical Computing,2008 Reference Source

[ref-20] Ranson H Jensen B Vulule JM: Identification of a point mutation in the voltage-gated sodium channel gene of Kenyan *Anopheles gambiae* associated with resistance to DDT and pyrethroids. *Insect Mol Biol.* 2000;9(5):491–497. 10.1046/j.1365-2583.2000.00209.x 11029667

[ref-21] Ranson H Lissenden N: Insecticide Resistance in African *Anopheles* Mosquitoes: A Worsening Situation that Needs Urgent Action to Maintain Malaria Control. *Trends Parasitol.* 2016;32(3):187–96. 10.1016/j.pt.2015.11.010 26826784

[ref-22] Reid MC McKenzie FE: The contribution of agricultural insecticide use to increasing insecticide resistance in African malaria vectors. *Malar J.* 2016;15:107. 10.1186/s12936-016-1162-4 26895980 PMC4759738

[ref-24] Santolamazza F Mancini E Simard F: Insertion polymorphisms of *SINE200* retrotransposons within speciation islands of *Anopheles gambiae* molecular forms. *Malar J.* 2008;7:163. 10.1186/1475-2875-7-163 18724871 PMC2546427

[ref-25] Tripet F Wright J Cornel A: Longitudinal survey of knockdown resistance to pyrethroid ( *kdr*) in Mali, West Africa, and evidence of its emergence in the Bamako form of *Anopheles gambiae s.s*. *Am J Trop Med Hyg.* 2007;76(1):81–7. 17255234

[ref-26] Wang L: Functional and pharmacological analysis of insect sodium channels in xenopus oocytes. A dissertation, Entomology - Doctor of Philosophy. Michigan State University,2013;3–166. Reference Source

[ref-27] WHO: World Malaria Report 2016. World Health Organization, Geneva;2016 Reference Source

[ref-28] WHO: Global Malaria programme 2016. Implications of insecticide resistance for malaria vector control. World Health Organization, Geneva;2016 Reference Source

[ref-29] WHO: Test Procedures for Insecticide Resistance Monitoring in Malaria Vectors. World Health Organization, Geneva;2013 Reference Source

