# Supplementary material for: First detection of N1575Y mutation in pyrethroid resistant Anopheles gambiae in Southern Côte d’Ivoire
Source: Wellcome Open Res. 2017 Aug 24;2:71. [Version 1] doi: 10.12688/wellcomeopenres.12246.1 (PMC5627500; doi:10.12688/wellcomeopenres.12246.1)
Supplement: Peer review history

# Peer review: Referee response for version 1

**DOI:** 10.21956/wellcomeopenres.13258.r25868

**Published:** 2017-09-29

**Authors:** Jacob Williams

**Affiliations:** 1 Tropical Public Health Practice LLC, Washington, DC, USA

The primary purpose of reporting on the presence of N1575Y mutation in pyrethroid resistant *Anopheles gambiae* – is well demonstrated. This study provides crucial additional information to the body of knowledge on the evolving scenario of insecticide resistance in the West Africa sub-region.  There are few issues requiring the attention of the authors:

1. Consider a slight modification of the title of the paper to indicate evaluation of *Anopheles gambiae* **sensu lato**, since both *An. coluzzii* and *An. gambiae s.s.,* were evaluated: 
  "First detection of N1575Y mutation in pyrethroid resistant *Anopheles gambiae* *(s.l.)*, in Southern Côte d’Ivoire"
2. While the mosquitoes tested were 2-5 days old and varies from the WHO recommendation of 3-5 days old test samples, in this case, the discrepancy does not present a significant problem since the objective of the study to detect the presence of the resistance gene mutation. However, comparison of phenotypic expression from this study (i.e. mortality rates from WHO bio-assay), with results obtained from other datasets elsewhere, may be constrained. This is because mortality rates have been shown to vary with the age of mosquito and the addition of 2-day old samples, will be a confounding factor. 
3. It is unclear if the mosquito samples evaluated in Elibou ( *n*=44, Table 1) were those surviving the WHO deltamethrin bio-assay, or a subsample from the additional 110 samples the authors indicated were “untested” but also evaluated. I am inclined to believe that the 44 mosquitoes evaluated for N1575Y, were survivals from the 91 samples tested via WHO bio-assay. Please clarify.

Authors indicate ongoing monitoring. They are encouraged to ensure robust concurrent evaluations of the progress/frequency of mutation and the resistance rate (phenotypic) among the various subspecies of *An. gambiae s.l.* in the area, to capture the very likely differences among the species.

I have read this submission. I believe that I have an appropriate level of expertise to confirm that it is of an acceptable scientific standard.

# Peer review: Referee response for version 1

**DOI:** 10.21956/wellcomeopenres.13258.r25343

**Published:** 2017-09-18

**Authors:** Kobié H. Toe

**Affiliations:** 1 National Center for Research and Training on Malaria, Ouagadougou, Burkina Faso

The paper looks interesting, clear and well written. The report of the N1575Y mutation, which now contribute to pyrethroid resistance although only reported in some countries. This paper is the proof that it could be spread also in other countries. However I have noticed that some useful details which could lead to better understanding and avoid confusion of the work were missing. Please see comments to author.

**Abstract**: In the method paragraph it said that three to five days old mosquitoes were used for the bioassays. Which contradict what is mentioned in **the Methods-WHO diagnostic bioassays **part where it is mentioned that two to five day old mosquitoes were used. It is confusing. Give the right age of mosquitoes used for the bioassays. If it was two to five days mosquitoes used, explained why, as the WHO procedure you have cited recommends to use three to five days old mosquitoes. I do not know which is right.

**Methods:**

 In Elibou untested mosquito were used for molecular analysis, and in Tiassalé surviving mosquito to deltamethrim exposure were used.

 In Elibou the frequency of 1014F has not been investigated, there is a specific reason for that? It is known that the 1014F is linked to 1575Y. The data of the 1014F should be interesting. Providing this data should be important.

**Table1**: in this table presenting the results the frequencies of the 1575Y mutation in Tiassalé (n= 92) and Elibou (n= 44), I am a bit confused. 110 untested mosquitoes from Elibou were used for molecular. Why in the table the total number of mosquitoes from Elibou was only 44? These mosquitoes are from those surviving deltamethrim exposure or are subset of the 110 untested mosquitoes used for molecular form and species identification.

 Of the most recent paper ( http://www.ncbi.nlm.nih.gov/pubmed/25766412), reporting the presence of the 1575Y mutation in Tengrela area in the Burkina Faso, of the closest area from Côte d’Ivoire where this mutation is reported with a frequency around 0.25- is not cited anywhere in the paper. It could be interesting to report to this paper, as it is one of the most recent talking also about this mutation.

 I have hope that the authors will continue the monitoring of the mutation as mentioned in the conclusion.

I have read this submission. I believe that I have an appropriate level of expertise to confirm that it is of an acceptable scientific standard.
